# Evaluating rice lipid content, yield, and quality in response to nitrogen application rate and planting density

**DOI:** 10.3389/fpls.2024.1469264

**Published:** 2024-11-15

**Authors:** Guangyi Chen, Congmei Li, Mingming Hu, Xingmei He, Hong Yang, Qiuqiu Zhang, Chaoyue Wu, Qiang Duan, Ligong Peng, Yao Zhang, Ziyu Li, Yuyuan Ouyang, Yan Lan, Tian Li

**Affiliations:** ^1^ College of Agronomy, Sichuan Agricultural University, Chengdu, China; ^2^ Crop Research Institute, Sichuan Academy of Agricultural Sciences, Chengdu, China; ^3^ School of Life Science and Engineering, Southwest University of Science and Technology, Mianyang, China

**Keywords:** rice, lipid content, nitrogen application rate, planting density, yield, quality

## Abstract

To investigate the effects of nitrogen (N) application rate and planting density (D) on the contents of lipid and free fatty acid, fatty acid composition, yield and quality of rice grain, a field experiment was conducted using Koshihikari (japonica) as experimental material from 2021 to 2022 with three N levels (90, 150 and 210 kg ha^-1^, denoted as N1, N2 and N3, respectively) and three transplanting densities (19.0 × 10^4^, 26.7 × 10^4^ and 40.0 × 10^4^ plants ha^-1^, denoted as D1, D2 and D3, respectively). The results showed that N application rate and planting density had highly significant impacts only on the contents of free fatty acid and saturated fatty acid, respectively. Increased N and planting density enhanced the contents of lipid (29.41 mg g^-1^) and free fatty acid (21.47%). The highest values were obtained under N3D3 increasing by 7.02% and 3.23 percentage points, respectively, compared to other treatments. No significant differences in lipid content were found among treatments, whereas free fatty acid exhibited significant differences. The unsaturated fatty acid content increased with increasing N but first decreased and then increased with increasing planting density, while saturated fatty acid content showed the opposite trend. Appropriate N level and planting density improved the relative chlorophyll content and net photosynthetic rate of rice flag leaves, as well as increased grain yield, effective panicle number and spikelete number per panicle, but decreased the seed setting rate. Under N2D2, the relative chlorophyll content and net photosynthetic rate remained relatively high throughout the grain filling stage, resulting in the highest grain yield, with increases of 43.87-47.03% compared to other treatments. A moderate N level improved the milling quality of rice, while increased planting density reduced it. However, both increased N and planting density reduced the appearance quality and cooking and eating quality of rice. Overall, the effects of increasing N application rate and planting density on enhancing rice lipid and free fatty acid contents were limited. A combination of 150 kg ha^-1^ N application rate and 26.7 × 10^4^ plants ha^-1^ was recommended for achieving relatively higher yield, lipid content and better grain quality.

## 1 Introduction

Rice (*Oryza sativa* L.) is among the most significant food crops globally, with a particularly prominent role in food production across Asia, including China (Huang et al., 2018). As the global population continues to grow and living standards improve, there is an increasing demand for higher rice yields, improved eating quality, and enhanced nutritional and health benefits. Although the lipid content in rice is relatively low, approximately 1-2.6% in brown rice and about 0.2-2% in polished rice (Zhang et al., 2019), lipids offer more energy compared to proteins and starches. Despite the substantial yield potential of rice grains, which can reach 7 tons per hectare, compared to soybeans at 2 tons per hectare, rice remains underutilized for oil production. Therefore, if the oil content of rice can be enhanced to exceed 5%, it could serve as a highly efficient alternative to soybean in terms of oil yield per unit area (Liu et al., 2024). Moreover, the fatty acids in rice grains are predominantly high-quality unsaturated fatty acids (UFAs), including oleic acid, linolenic acid, and linoleic acid. These UFAs possess significant nutritional value and health benefits, such as the prevention of arteriosclerosis and hypercholesterolemia (Liu et al., 2013). The bioactive compounds found in rice bran oil, including γ-oryzanol, phytosterols, vitamin E, and squalene, have been shown to significantly lower blood lipids and serum cholesterol, improve neurological function, and provide antioxidant, anti-inflammatory, and anticancer benefits (Sharif et al., 2014).

N application rate and planting density are two critical agronomic methods in rice cultivation. Among these, N is the most sensitive factor affecting rice growth, development, and yield, directly influencing physiological processes such as photosynthesis and grain filling (Tang et al., 2020). Planting density, on the other hand, is fundamental for optimizing the population structure, enhancing light energy utilization, and thereby increasing yield (Li, 2020). Substantial progress has been made in studying N application rate and planting density in rice production in Southwest China. The N application rate in this region typically averages around 167 kg ha^-1^, slightly below the national average (Huang et al., 2020). Extensive research has been conducted on the impact of these two factors on rice yield and quality formation. Numerous studies indicate that, within a certain range, the N application rate and planting density exhibit a parabolic relationship with yield. A significant interaction exists between these two factors, leading to an optimal combination for achieving high rice yields (Chen et al., 2014a; Zhou et al., 2013). Moreover, moderate N application rate and planting density can enhance the nutritional and eating quality of hybrid japonica rice (N: 141.75 kg ha^-1^, D: 51.00 × 10^4^ plants ha^-1^) and conventional japonica rice (N: 150 kg ha^-1^, D: 60 kg ha^-1^) (Chen et al., 2014b; Cheng et al., 2011). Under low planting density conditions (180.00 ×10^4^ plants ha^-1^), increasing N application rate can improve milling quality. However, under high planting density conditions (360.00 ×10^4^ plants ha^-1^), increasing N application rate has no significant effect on the milled rice rate (MR) and head rice rate (HR). The impact of planting density on chalkiness degree (CD) interacts with N fertilizer rate, at high N levels (300 kg ha^-1^), increasing planting density can increase CD (Chen et al., 2010a, 2010).

Research has demonstrated that N application rates significantly influence the lipid content and fatty acid composition ratios of crops. In maize, lipid content first increases and then decreases with rising N application rates (0-337.50 kg ha^-1^) (Tai, 2010). For soybeans, lipid content at maturity stage increases with higher N application rates (0-300.00 kg ha^-1^) (Gong, 2010). In brown rice, late-stage N application (flower-promoting fertilizers) significantly reduces lipid content, with the rate of reduction slowing as N levels increase (0-225.00 kg ha^-1^) (Zhu et al., 2006). N application (0-180.00 kg ha^-1^) can first increase and then decrease lipid content in rice with high anthocyanin content (Shi, 2017). Japonica rice exhibits significantly higher lipid content under medium nitrogen levels (180 kg ha^-1^) compared to other treatments (Gu, 2011). N fertilizer can enhance the activity of key enzymes involved in lipid synthesis, thereby promoting the accumulation of lipid and free fatty acid (FFA), and optimizing the ratio of unsaturated to saturated fatty acids (SFAs) (Tu et al., 2020). In comparison, limited research can be found on the impact of planting density on crop lipid content. Some studies indicate that appropriately increasing planting density enhances phosphatidate phosphatase and glucose-6-phosphate dehydrogenase activities in rapeseed seeds, promoting lipid metabolism and thus increasing lipid content (Tang and Guan, 2001). However, other research shows that increased planting density reduces lipid content in maize grains while significantly increasing protein content (Rafiq et al., 2010). In cotton seeds, both biomass and lipid content significantly decrease with higher planting densities during development and at maturity stage (Zhu et al., 2016).

Although previous studies have provided valuable insights into the effects of N application rate and planting density on rice yield and quality, the interaction between these factors and their impact on lipid content, FFA content, and fatty acid composition in rice grain remains underexplored. The objectives of this study were to investigate the effects of nitrogen application rate and planting density on rice lipid content, yield and quality, and by which the results. The findings were aimed to provide theoretical references for optimizing high-yield and high-quality rice cultivation methods in the terms of increase of lipid content and fatty acid composition.

## 2 Materials and methods

### 2.1 Experimental site and materials

**Figure 1:**
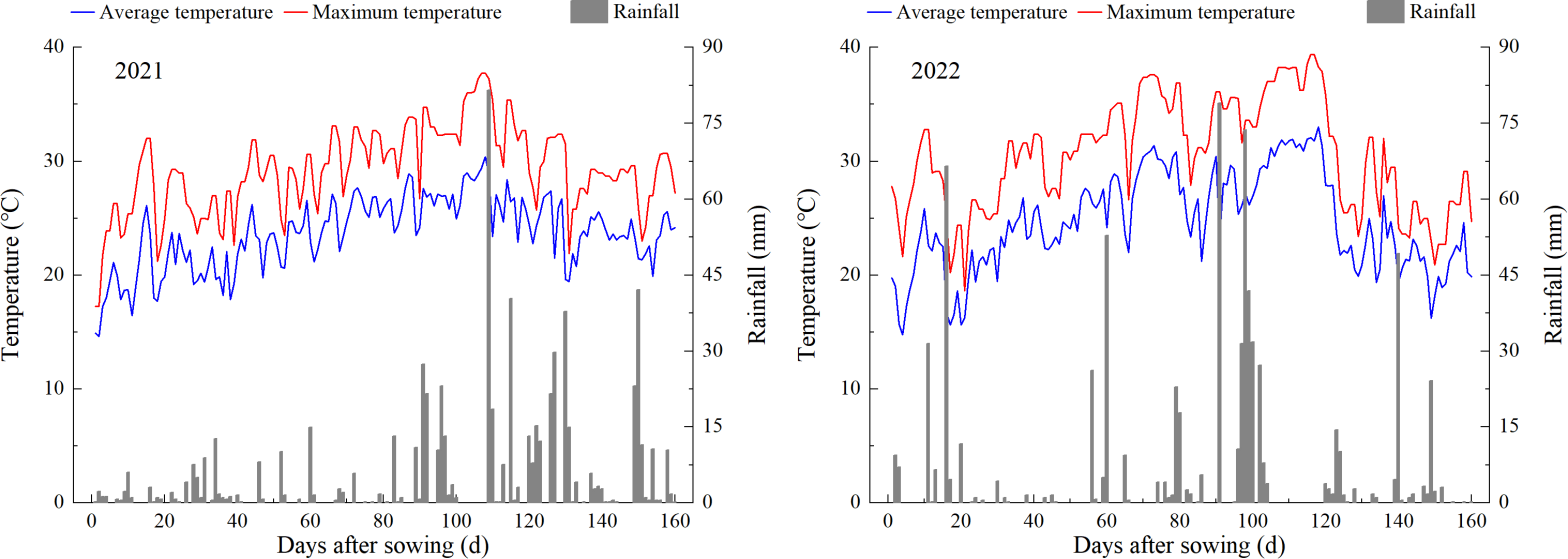
Climate data during the experimental periods.

Field experiments were conducted during the growing seasons of 2021 and 2022 at the research farm of Sichuan Agricultural University, Wenjiang city, Sichuan Province, China (30°43′ N, 103°47′ E). The field soil was a sandy loam. Prior to the establishment of the field experiment, soil samples from the topsoil layer (0-0.20 m) were analyzed. The climate data and analysis results of the top soil layer were shown in **Table 1** and **Figure 1**, respectively. During rice growth period in two years, the daily average temperature and rainfall were 23.66°C and 4.24 mm in 2021, and 24.59°C and 4.66 mm in 2022, respectively. The high-yield and high-quality japonica rice cultivar Koshihikari (Kyushu University and Agricultural Biological Resources Research Institute of Japan), with a total growth period of approximately 135-138 days, was chosen and used as the test material.

**Table 1:** Soil properties of the top soil layer (0-0.20 m) at the experimental sites.

Years	pH	Organic matter (g kg^-1^)	Total N (g kg^-1^)	Available N (mg kg^-1^)	Available P (mg kg^-1^)	Available K (mg kg^-1^)
2021	5.97	33.39	2.04	125.70	35.01	60.93
2022	5.88	29.75	1.91	110.53	26.28	57.95

N, P, K represent nitrogen, phosphorus and potassium, respectively.

### 2.2 Experimental design

An orthogonal experimental design was adopted with two factors in two years, using a randomized complete block design with three replicates. Nine treatments were established by the complete combination of three N application rates (90, 150 and 210 kg ha^-1^, denoted as N1, N2 and N3, respectively) and three planting densities (19.0 × 10^4^, 26.7 × 10^4^ and 40.0 × 10^4^ plants ha^-1^, denoted as D1, D2 and D3, respectively).

Seeds were sown on 18 April 2021 and 28 April 2022, and the seedlings were transplanted on 25 May 2021 and 1 Jun 2022, and rice was harvested on 14 Sept 2021 and 15 Sept 2022, respectively. The area of each test plot was 5.0 m×5.0 m, and the transplanting density (row spacing × plant spacing) was at the treatment densities specified above (also regarded as 35 cm × 30 cm, 30 cm × 25 cm and 25 cm × 20 cm, denoted as D1, D2 and D3, respectively) with two seedlings per hill. Urea (N, 46.4%) was used as the N source, superphosphate (P_2_O_5_, 12.0%) was used as the phosphorus (P) source, and potassium chloride (K_2_O, 60.0%) was used as the K source. N fertilizer was used as base fertilizer, tillering fertilizer, and panicle fertilizer at a 3:3:4 ratio. Basal N (at the treatment rates specified above), P (90 kg ha^-1^) and K (180 kg ha^-1^) were applied to the soil one day before transplanting. For the fertilizer treatments, ridges with plastic film were used for separation, and protection lines were established between the treatment blocks to ensure the isolation of the experimental plots. During the whole growing season, rice was continuously flooded with the river water. Field management, including the prevention and control of pests and weeds, was conducted according to the local cultural practices.

### 2.3 Measurements and methods

10 hills of plants with the same growth were selected 7 d (early filling stage), 21 d (middle filling stage) and 36 d (late filling stage) for the measurements of relative chlorophyll content (SPAD value) and net photosynthetic rate (Pn). At harvest, 10 hills of plants from each plot were sampled randomly and allowed to dry naturally in the sun to assess the rice yield and yield components, milling quality and appearance quality after the material was stored at room temperature for 3 months. The milled rice was crushed and sieved through a 100-mesh screen for measurements of the lipid content, fatty acid content, fatty acid composition and (rapid visco-analyzer) RVA profile characters.

#### 2.3.1 Relative chlorophyll content and net photosynthetic rate

The relative chlorophyll content of flag leaf was measured using a portable chlorophyll meter (SPAD-502, Konica Minolta Holdings Inc., Tokyo, Japan). Six points on each leaf were chosen fro measurement with SPAD. These comprised three pairs of points on both sides of the midrib were sampled near the leaf tip, in the middle of the leaf, and near the leaf bottom. The mean of these six SPAD values was recorded.

The net photosynthetic rate of flag leaf was measured using a photosynthetic instrument (GFS-3000, Zealquest Scientific Technology Co., Ltd., Shanghai, China) around 9:00 a.m to 11:00 a.m under sunny conditions. The chamber settings held constant across measurements were: 1200 µmol m^-2^ s^-1^ irradiance, 30°C air temperature and 400 µmol mol^-1^ CO_2_ concentration.

#### 2.3.2 Lipid and fatty acid contents, and fatty acid composition

Lipids in the rice grains were obtained by ultrasound-assisted extraction (UAE) according to a previously reported method with a slight modification (Tu et al., 2020). UAE was carried out with an ultrasonic cleaner (CPX3800H-C, Emerson Electric Co., St. Louis, MO, USA). Samples (2.000 g each) were weighed and extracted with 50 mL of n-hexane in a centrifuge tube and then mixed thoroughly using a Vortex Genie (G560E, Scientific Industries Inc., Bohemia, NY, USA). The tube was immersed in an ultrasonic cleaner bath, and the lipids were extracted with the appropriate sonication power (110 W), duration (37 min) and temperature (42°C). The supernatant was transferred to a centrifuge tube and centrifuged (5430R, Eppendorf AG, Hamburg, Germany) for 10 min at 7000 r min^-1^. The supernatant was then transferred to a conical flask (weighed and recorded as m_1_ beforehand) and evaporated using an electric hot plate (ML-3-4, Beijing Zhongxing Weiye Century Instrument Co., Ltd., Beijing, China), and the sample was ultimately dried and weighed (recorded as m_2_). The lipid content (m_3_) in the rice grain was then calculated using the following formula: m_3_ = m_2_ - m_1_.

The free fatty acid content in the UAE samples was measured according to a previously reported method (Tu et al., 2020). The extracted lipids and 100 mL of diethyl ether/95% ethanol (v:v, 1:1) were mixed together, and three drops of phenolphthalein indicator were added to the mixture. Then, each mixture was titrated with 0.1 mol L^-1^ KOH solution until the color changed to red, and the volume of KOH solution used was recorded. The FFA content in rice grain was calculated using the following formula: ω = V × C × 282 × 100%/1000m, where ω is the FFA content (%), V is the volume of KOH (mL), C is the concentration of KOH (mol L^-1^), 282 is the molar mass of oleic acid (g mol^-1^), and m is the lipid content in the rice grain (g).

The fatty acid composition in the UAE samples were measured by using a gas chromatograph-mass spectrometer (GC-MS) according to a previously reported method (Chen et al., 2023). The extracted lipids and 1 mL of petroleum ether/benzene (v:v, 1:1) were mixed together and then flushed with 1 mL of petroleum ether/benzene. All the liquid phases were collected and transferred into a 10 mL volumetric flask. The samples were neutralized with 0.4 mol L^-1^ KOH/carbinol, mixed thoroughly, incubated at room temperature for 10 min, and then brought to volume by the addition of water, followed by mixing. The organic layer was collected for analysis via GC-MS (Agilent 7890-5975C, Agilent Technologies Co., Ltd., Santa Clara, CA, USA) and the NIST Mass Spectral Database (National Institute of Standards and Technology, Gaithersburg, MD, USA). The GC-MS conditions and components were as follows: carrier gas (He), approximately 1 mL min^-1^; oven temperature: initially maintained at 80°C for 3 min, increased at 10 °C min^-1^ to 260°C, and then held for 15 min; injected sample volume, 1 μL; injection, split 100:1; and mass ranges, 20-500 m/z. Fatty acids were identified by comparisons with fatty acid standards. The masses of fatty acid methyl esters were quantified as percentages of the total methyl ester peak area.

#### 2.3.3 Rice yield and yield components

Rice was harvested at maturity stage and the yield in each treatment was recorded after measuring moisture content and removing impurities. Rice yield was adjusted to a moisture content of 14%. The effective panicle number (PN) was determined before harvest using 30 hills of plants per plot, and 10 hills of plants were selected from each plot according to the average number of effective panicles. The spikelete number per panicle (SP), 1000-grain weight (GW), seed setting rate (SR), and grain yield (GY) were determined.

#### 2.3.4 Milling, appearance, cooking and eating qualities, and rapid visco-analyzer

About 130.0 g rice grains were processed by using a rice huller (JLG-2118, Taizhou Food Instrument Co., Ltd., Zhejiang, China) to obtain brown rice. The brown rice was polished by using a rice milling machine (JNMJ-3, Taizhou Food Instrument Co., Ltd., Zhejiang, China) to obtain milled rice. In order to obtain head-milled rice, grain with a length longer than 3/4 of its total length was separated from the milled rice by using a broken rice separator (FQS-13X20, Taizhou Food Instrument Co., Ltd., Zhejiang, China). The brown rice, milled rice, and head-milled rice are expressed as percentages of the total grain weight.

The chalkiness rate (CR) and chalkiness degree were determined using a grain appearance analyzer (JMWT12, Dongfu Jiuheng Instrument Technology Co., Ltd., Beijing, China).

The sensory properties of the cooked rice were measured using a rice sensory analyzer (STA 1B, Satake Asia Co., Ltd., Tokyo, Japan). Milled rice (30.00 g) was washed in a stainless-steel container and then transferred into a 50 mL aluminum box containing 40 mL of water. The milled rice was cooked in a multifunctional, timed food steamer (GF-339, Goodway Electrical Enterprise Ltd., Hong Kong, China). After the cooking, the sensory properties of the cooked rice were determined. Cooked rice texture properties were measured using a rice texture analyzer (RHS 1A, Satake Asia Co., Ltd., Tokyo, Japan).

A 3.00 g sample and 25.0 mL of distilled water were added to a test tube. Pasting properties were measured by using a rapid visco-analyzer device (3-D, Newport Scientific, Sydney, Australia) and analyzed with Thermal Cycle for Windows software. Viscosity values were measured in a rapid viscosity analyzer unit (RVU).

### 2.4 Statistical analysis

Data were analyzed using analysis of variance (ANOVA) after checking the assumptions of homogeneity of variances and normality, and means were compared based on the least significant difference (LSD) test at the 0.05 probability level using SPSS 23.0 (Statistical Product and Service Solutions Inc., Chicago, IL, USA). Origin Pro 2020 (OriginLab, Northampton, MA, USA) was used to do the correlation analysis and draw the figures. All the data presented in the Results section represent findings from a two-year study, with the exception of the fatty acid composition data, which was measured specifically in 2021.

## 3 Results

### 3.1 Rice physiological characteristics

**Figure 2:**
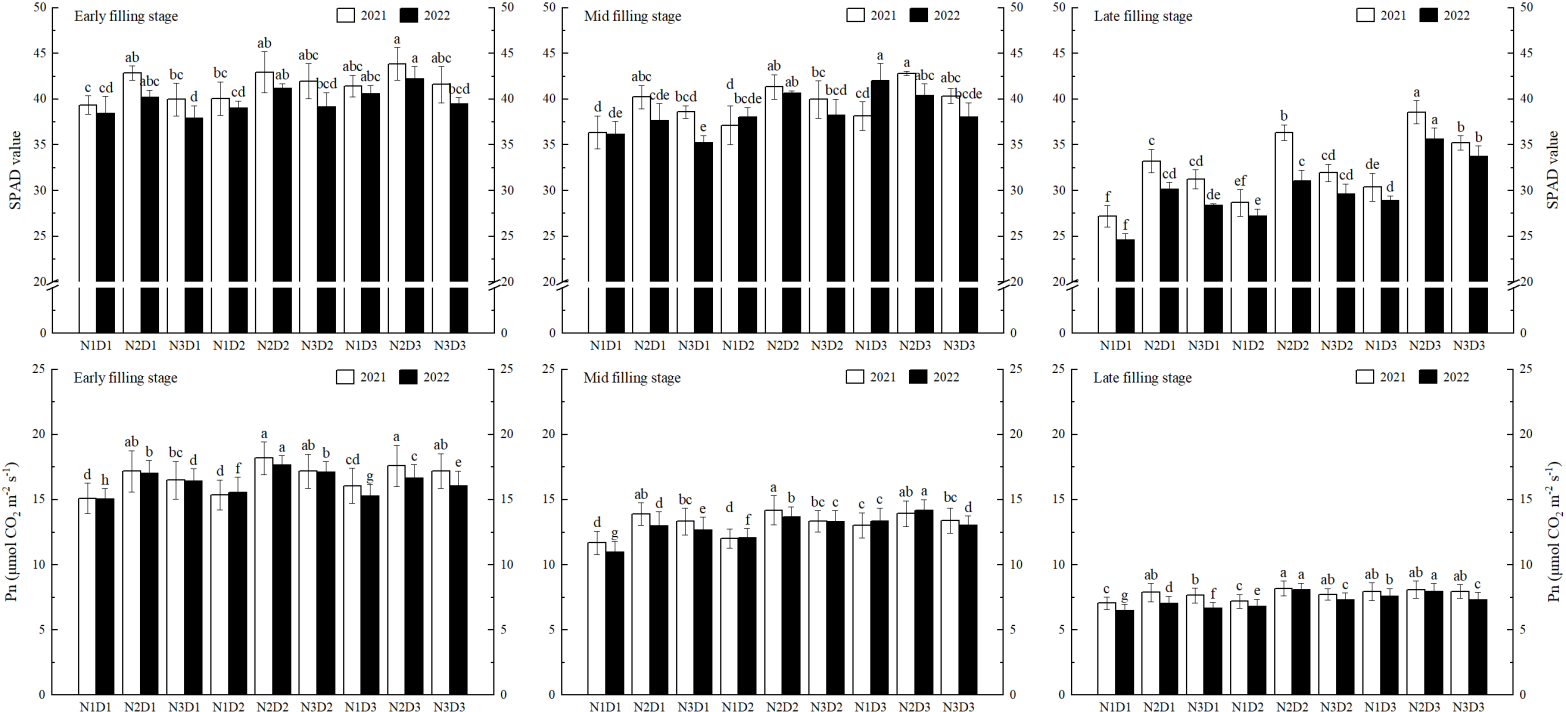
Effects of nitrogen application rate and planting density on flag leaf SPAD value and Pn. SPAD and Pn represent relative chlorophyll content and net photosynthetic rate, respectively. N1, N2 and N3 refer to the different nitrogen fertilizer treatments (90, 150 and 210 kg ha^-1^, respectively). D1, D2 and D3 refer to the different planting density treatments (19.0 × 10^4^, 26.7 × 10^4^ and 40.0 × 10^4^ plants ha^-1^, respectively). Different lowercase letters in the same color column mean the significant difference between treatments at *p* < 0.05. The data presented are the mean ± standard deviation, *n* = 3.

Increasing N application rate and planting density could enhance the SPAD value and Pn of rice flag leaves within a certain range. The impact of N application on improving SPAD value and Pn was more pronounced than that of planting density (**Figure 2**).

During the grain-filling process, the SPAD value and Pn of rice flag leaves gradually decreased. With increasing N application rate and planting density, both SPAD value and Pn exhibited an increasing or initially increasing and then decreasing trend (N1 < N3 < N2, D1 < D3 < D2).

**Figure 3:**
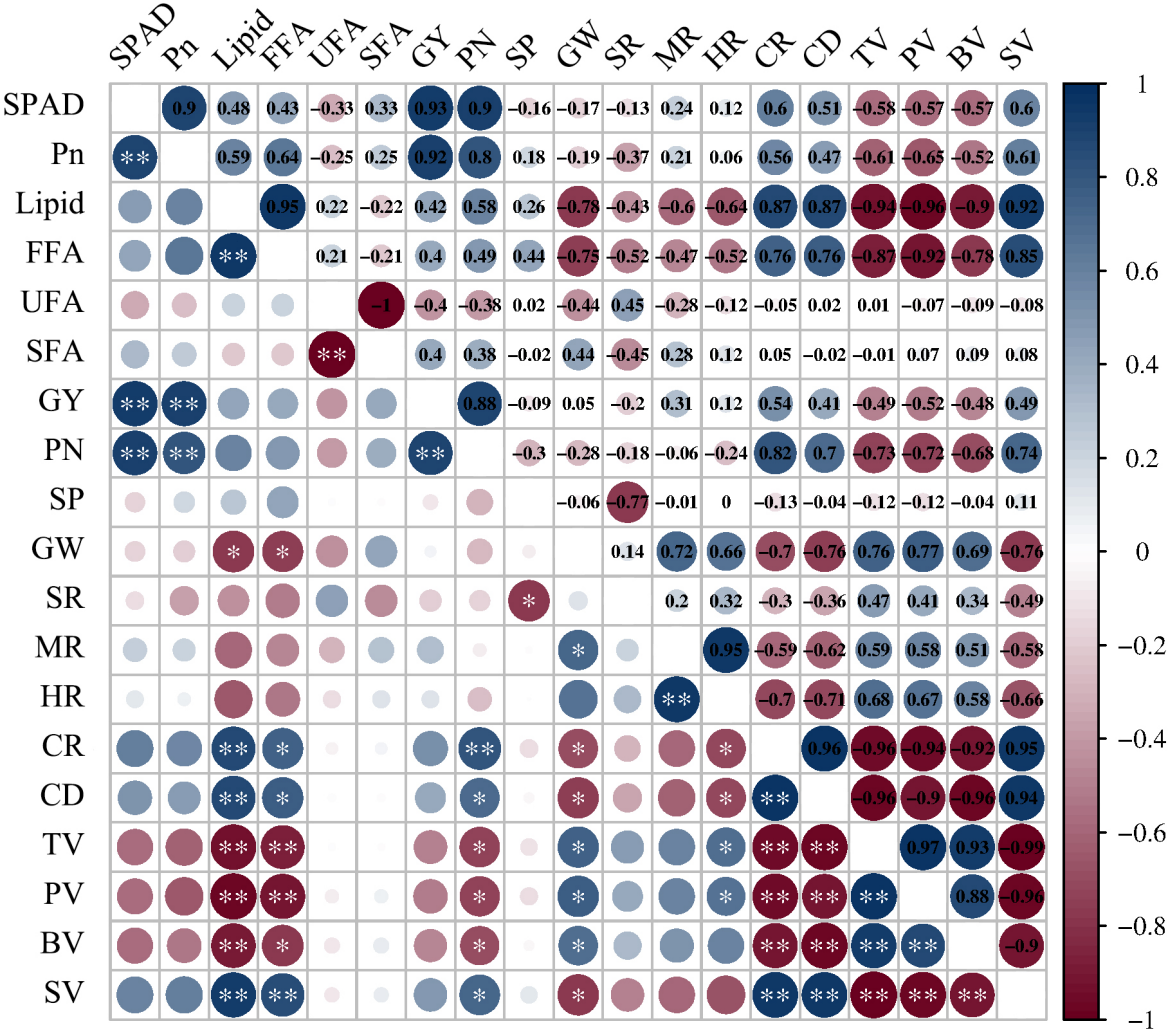
Correlation coefficients of physiological characteristics and lipid content with rice yield, yield components and rice quality. SPAD, Pn, FFA, UFA, SFA, GY, PN, SP, GW, SR, MR, HR, CR, CD, TV, PV, BV and SV represent relative chlorophyll content, net photosynthetic rate, free fatty acid, unsaturated fatty acid, saturated fatty acid, grain yield, effective panicle number, spikelet number per panicle, 1000-grain weight, seed setting rate, milled rice rate, head rice rate, chalkiness rate, chalkiness degree, taste value, peak viscosity, breakdown viscosity and setback viscosity, respectively. ANOVA *p* values and symbols were defined as: * *p* < 0.05; ** *p* < 0.01; ns: *p* > 0.05.

Variance analysis indicated that N application rate and planting density had highly significant effects on the SPAD value and Pn of rice flag leaves (**Table 2**). Additionally, there was a significant interaction effect between N application rate and planting density on the Pn. Under N2D3 or N2D2, SPAD value and Pn remained at relatively high levels throughout the grain-filling stage. Compared to other treatments, the SPAD value and Pn increased by 20.49% and 20.54%, respectively. Correlation analysis showed that SPAD value and Pn were significantly positively correlated with GY and PN (**Figure 3**), indicating that higher SPAD values and Pn had greater yield-increasing potential.

**Table 2:** Analysis of variance on SPAD value, Pn, and the contents of lipid, free fatty acid, unsaturated fatty acid and saturated fatty acid.

ANOVA	SPAD	Pn	Lipid	FFA	UFA	SFA
Year (Y)	43.043**	11.304**	7.287*	7.596**	–	–
Nitrogen (N)	64.456**	48.741**	3.281ns	19.046**	0.150ns	0.990ns
Density (D)	42.929**	10.589**	0.332ns	0.494ns	0.785ns	5.172*
Y × N	6.232**	0.342ns	0.027ns	0.006ns	–	–
Y × D	1.121ns	0.768ns	0.003ns	0.151ns	–	–
N × D	0.169ns	2.710*	0.045ns	0.735ns	0.010ns	0.065ns
Y × N × D	0.186ns	0.241ns	0.027ns	0.032ns	–	–

SPAD, Pn, FFA, UFA and SFA represent relative chlorophyll content, net photosynthetic rate, free fatty acid, unsaturated fatty acid and saturated fatty acid, respectively. ANOVA *p* values and symbols were defined as: * *p* < 0.05; ** *p* < 0.01; ns: *p* > 0.05. The data presented are the mean ± standard deviation, *n* = 3.

### 3.2 Lipid content and fatty acid composition

**Figure 4:**
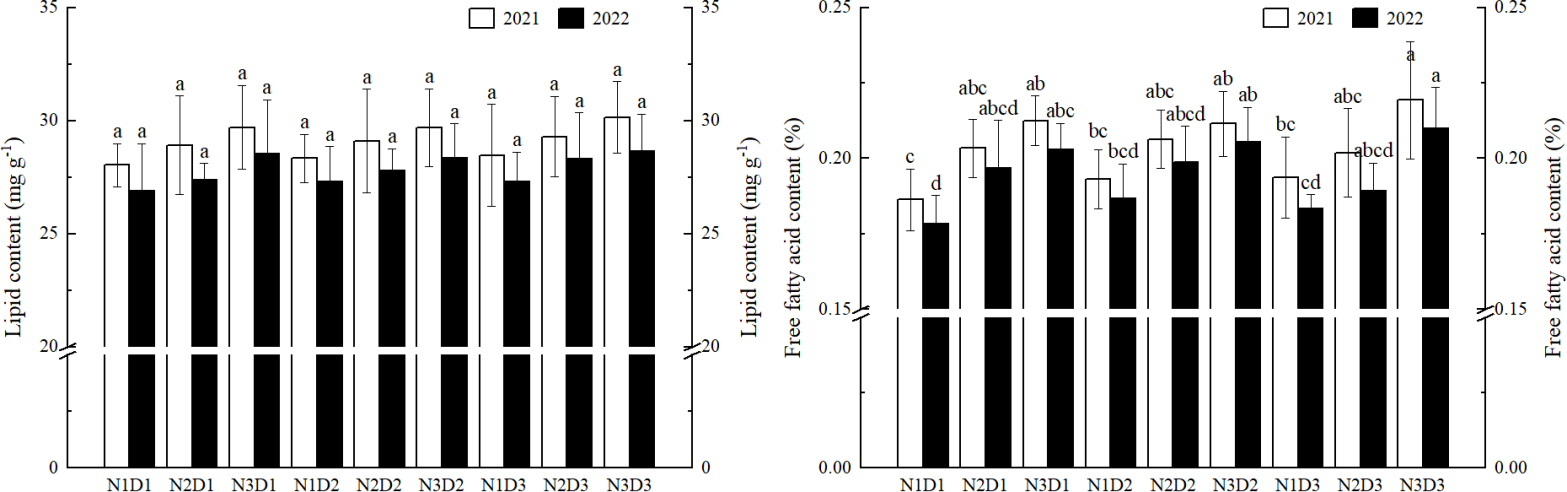
Effects of nitrogen application rate and planting density on lipid and free fatty acid contents in rice grain. N1, N2 and N3 refer to the different nitrogen fertilizer treatments (90, 150 and 210 kg ha^-1^, respectively). D1, D2 and D3 refer to the different planting density treatments (19.0 × 10^4^, 26.7 × 10^4^ and 40.0 × 10^4^ plants ha^-1^, respectively). Different lowercase letters in the same color column mean the significant difference between treatments at *p* < 0.05. The data presented are the mean ± standard deviation, *n* = 3.

The contents of lipid and FFA in rice grain increased with higher N application rates and planting densities (**Figure 4**). As more N was gradually applied, both lipid and FFA contents increased. However, with the increase in planting density, the FFA content did not follow a clear pattern. Under low and medium N levels, lipid content increased with increasing planting density, while it first decreased and then increased under high N levels (D3 > D1 > D2). The highest contents of lipid (29.41 mg g^-^¹) and FFA (21.47%) were observed under N3D3. No significant differences were found in lipid content among the various treatments, while differences in FFA content were significant. Compared to other treatments, the N3D3 treatment resulted in the highest increases in lipid and FFA contents by 7.02% and 3.23 percentage points, respectively.

A total of 14 fatty acid components were detected in the rice cultivar Koshihikari, including 6 UFAs and 8 SFAs (**Table 3**). The highest contents were of palmitic acid (C16:0), linoleic acid (C18:2), oleic acid (C18:1), and stearic acid (C18:0), which together accounted for over 90% of the total fatty acid content. As more N was gradually applied, the content of UFAs increased, while the SFA showed the opposite trend. With increasing planting density, the content of UFA first decreased and then increased (D1 > D3 > D2), whereas the content of SFA first increased and then decreased (D1 < D3 < D2). Under N3D1, the highest content of UFA (72.99%) and the lowest content of SFA (27.01%) were observed, while the lowest content of UFA (70.10%) and the highest content of SFA (29.90%) were found under N1D2. No significant differences were found in UFA content among the various treatments, while differences in SFA content were significant.

**Table 3:** Effects of nitrogen application rate and planting density on fatty acid composition in rice grain (%).

Fatty acid composition	N1D1	N2D1	N3D1	N1D2	N2D2	N3D2	N1D3	N2D3	N3D3
Lauric acid, C12:0	0.40 ± 0.04e	0.58 ± 0.03a	0.42 ± 0.04de	0.50 ± 0.04bc	0.49 ± 0.02bc	0.53 ± 0.02abc	0.57 ± 0.04a	0.47 ± 0.04cd	0.54 ± 0.01ab
Myristic acid, C14:0	0.26 ± 0.02ab	0.25 ± 0.02b	0.24 ± 0.01b	0.25 ± 0.02b	0.26 ± 0.01ab	0.27 ± 0.02ab	0.29 ± 0.02a	0.26 ± 0.02ab	0.25 ± 0.02ab
Palmitoleic acid, C16:1	0.16 ± 0.01bc	0.14 ± 0.01d	0.16 ± 0.01bc	0.17 ± 0.01ab	0.15 ± 0.01cd	0.11 ± 0.01e	0.13 ± 0.01d	0.15 ± 0.01cd	0.19 ± 0.01a
Palmitic acid, C16:0	21.33 ± 0.97a	21.26 ± 0.86a	21.08 ± 0.93a	23.28 ± 2.24a	23.22 ± 1.21a	22.84 ± 1.59a	22.41 ± 2.13a	22.33 ± 0.72a	21.64 ± 1.45a
Linolenic acid, C18:3	0.33 ± 0.02d	0.38 ± 0.03cd	0.45 ± 0.04ab	0.41 ± 0.04bc	0.42 ± 0.03abc	0.34 ± 0.02d	0.47 ± 0.04a	0.38 ± 0.01cd	0.42 ± 0.03abc
Linoleic acid, C18:2	32.02 ± 1.95a	33.77 ± 2.04a	35.68 ± 3.07a	32.09 ± 2.35a	32.67 ± 2.47a	33.84 ± 2.47a	32.32 ± 2.2a	33.14 ± 1.34a	33.74 ± 2.35a
Oleic acid, C18:1	39.27 ± 2.81a	37.61 ± 2.91a	36.36 ± 2.48a	37.09 ± 2.50a	36.51 ± 1.56a	35.83 ± 1.93a	37.26 ± 1.31a	36.81 ± 1.95a	37.14 ± 3.44a
Stearic acid, C18:0	3.15 ± 0.16ab	2.89 ± 0.23bc	2.69 ± 0.22c	3.27 ± 0.17a	3.21 ± 0.16ab	3.06 ± 0.28ab	3.31 ± 0.09a	3.10 ± 0.13ab	2.88 ± 0.19bc
Gadoleic acid, C20:1	0.35 ± 0.02c	0.37 ± 0.02c	0.35 ± 0.02c	0.34 ± 0.03c	0.39 ± 0.03bc	0.48 ± 0.02a	0.44 ± 0.02ab	0.38 ± 0.02c	0.45 ± 0.02a
Arachidic acid, C20:0	0.72 ± 0.06bcde	0.67 ± 0.03de	0.66 ± 0.05e	0.68 ± 0.05de	0.75 ± 0.04abcd	0.77 ± 0.05abc	0.82 ± 0.07a	0.80 ± 0.03ab	0.70 ± 0.05cde
Heneicosanoic acid, C21:0	0.48 ± 0.02a	0.36 ± 0.02de	0.38 ± 0.01cd	0.42 ± 0.03bc	0.33 ± 0.02e	0.39 ± 0.02cd	0.36 ± 0.02de	0.41 ± 0.04c	0.46 ± 0.01ab
Docosanoic acid, C22:0	0.56 ± 0.04b	0.65 ± 0.03a	0.45 ± 0.02c	0.54 ± 0.02b	0.67 ± 0.05a	0.56 ± 0.02b	0.67 ± 0.05a	0.65 ± 0.03a	0.65 ± 0.03a
Tetracosanoic acid, C24:0	0.51 ± 0.04bcd	0.48 ± 0.04d	0.64 ± 0.03a	0.42 ± 0.02e	0.50 ± 0.02cd	0.54 ± 0.04b	0.48 ± 0.02d	0.51 ± 0.04bc	0.43 ± 0.02e
Hexacosanoic acid, C26:0	0.45 ± 0.04e	0.59 ± 0.03ab	0.45 ± 0.02de	0.54 ± 0.02bc	0.45 ± 0.03e	0.43 ± 0.02e	0.47 ± 0.04de	0.61 ± 0.04a	0.51 ± 0.04cd
UFA	72.13 ± 4.76a	72.27 ± 3.34a	72.99 ± 4.1a	70.10 ± 1.52a	70.13 ± 2.19a	70.61 ± 3.55a	70.62 ± 2.72a	70.86 ± 2.29a	71.94 ± 5.66a
SFA	27.87 ± 0.95ab	27.73 ± 0.81ab	27.01 ± 0.96b	29.90 ± 2.20a	29.87 ± 1.27a	29.39 ± 1.93ab	29.38 ± 2.14ab	29.14 ± 0.80ab	28.06 ± 1.26ab

N1, N2 and N3 refer to the different nitrogen fertilizer treatments (90, 150 and 210 kg ha^-1^, respectively). D1, D2 and D3 refer to the different planting density treatments (19.0 × 10^4^, 26.7 × 10^4^ and 40.0 × 10^4^ plants ha^-1^, respectively). UFA and SFA represent unsaturated fatty acid and saturated fatty acid, respectively. Different lowercase letters followed the values in the same line mean the significant difference between treatments at *p* < 0.05. The data presented are the mean ± standard deviation, *n* = 3.

Variance analysis showed that N application rate had a highly significant effect on the content of FFA, while planting density had a highly significant effect on the content of SFA (**Table 2**).

### 3.3 Rice yield and yield components

Increasing N application rate and planting density could increase GY, PN and SP, but reduced SR. The pattern of changes in GW varied under different N application rate and planting density levels. A moderate planting density combined with a medium N level could significantly increase GY (**Table 4**).

**Table 4:** Effects of nitrogen application rate and planting density on rice yield and yield components.

Year	Treatment	PN (×10^4^ ha^-1^)	SP	GW (g)	SR (%)	GY (kg ha^-1^)
2021	N1D1	233.33 ± 22.19d	98.00 ± 2.00de	24.70 ± 0.10b	93.12 ± 2.61a	5254.49 ± 442.68e
	N2D1	284.67 ± 17.04bc	104.33 ± 3.51bc	23.52 ± 0.08d	91.02 ± 3.09ab	6350.88 ± 307.11c
	N3D1	275.33 ± 30.66bcd	108.33 ± 4.16ab	22.93 ± 0.03f	88.76 ± 4.39abc	6052.89 ± 535.85cd
	N1D2	263.33 ± 24.01cd	101.00 ± 3.46cd	24.39 ± 0.07c	85.17 ± 1.82cd	5509.57 ± 231.24de
	N2D2	337.67 ± 20.79a	109.00 ± 3.61ab	25.23 ± 0.11a	81.54 ± 2.17de	7559.43 ± 130.49a
	N3D2	320.33 ± 24.58ab	111.00 ± 2.00a	22.86 ± 0.08f	77.45 ± 2.85e	6295.04 ± 546.50c
	N1D3	336.67 ± 31.09a	87.00 ± 1.73f	24.46 ± 0.09c	93.92 ± 1.95a	6717.81 ± 459.40bc
	N2D3	364.33 ± 27.65a	93.67 ± 2.52e	23.35 ± 0.07e	90.79 ± 1.50ab	7220.93 ± 240.34ab
	N3D3	352.67 ± 19.66a	98.00 ± 2.65de	22.61 ± 0.09g	87.20 ± 3.51bc	6812.83 ± 513.06abc
2022	N1D1	230.67 ± 15.04e	95.67 ± 5.13bcd	22.60 ± 0.16b	89.20 ± 4.73ab	4440.63 ± 299.79d
	N2D1	280.33 ± 18.56cd	102.00 ± 6.00abc	21.38 ± 0.11d	87.53 ± 3.69ab	5355.77 ± 594.89bc
	N3D1	271.00 ± 22.61d	105.00 ± 5.57ab	20.85 ± 0.13e	84.41 ± 5.45abc	5001.42 ± 503.66cd
	N1D2	259.00 ± 16.70de	106.67 ± 6.66a	22.04 ± 0.08c	81.54 ± 4.07bcd	4956.05 ± 358.11cd
	N2D2	333.33 ± 30.99ab	108.00 ± 4.36a	23.18 ± 0.15a	78.32 ± 4.33cd	6528.99 ± 639.41a
	N3D2	316.00 ± 24.27bc	108.67 ± 6.66a	20.54 ± 0.20f	74.86 ± 3.88d	5269.81 ± 426.06c
	N1D3	332.33 ± 23.76ab	84.00 ± 4.58e	22.01 ± 0.08c	91.05 ± 2.41a	5589.16 ± 442.22bc
	N2D3	362.00 ± 23.26a	91.67 ± 5.13de	21.16 ± 0.07d	87.83 ± 3.90ab	6149.11 ± 148.87ab
	N3D3	348.33 ± 20.79ab	94.33 ± 3.06cd	20.27 ± 0.09g	83.67 ± 2.48abc	5571.07 ± 372.25bc
F-value	Year (Y)	0.371ns	1.762ns	5748.368**	11.908**	72.448**
	Nitrogen (N)	22.700**	18.896**	1211.782**	13.594**	31.291**
	Density (D)	60.588**	61.923**	207.814**	39.708**	22.167**
	Y × N	0.001ns	0.607ns	2.990ns	0.008ns	0.496ns
	Y × D	0.001ns	0.979ns	4.601*	0.071ns	0.500ns
	N × D	1.723ns	0.483ns	224.020**	0.251ns	4.283**
	Y × N × D	0.003ns	0.445ns	1.379ns	0.030ns	0.154ns

N1, N2 and N3 refer to the different nitrogen fertilizer treatments (90, 150 and 210 kg ha^-1^, respectively). D1, D2 and D3 refer to the different planting density treatments (19.0 × 10^4^, 26.7 × 10^4^ and 40.0 × 10^4^ plants ha^-1^, respectively). PN, SP, GW, SR and GY represent effective panicle number, spikelet number per panicle, 1000-grain weight, seed setting rate and grain yield, respectively. Different lowercase letters followed the values in the same column mean the significant difference between treatments at *p* < 0.05. ANOVA *p* values and symbols were defined as: * *p* < 0.05; ** *p* < 0.01; ns: *p* > 0.05. The data presented are the mean ± standard deviation, *n* = 3.

As more N was gradually applied, PN first increased and then decreased (N2 > N3 > N1), SP increased, and SR decreased. With increasing planting density, PN increased, SP first increased and then decreased (D2 > D1 > D3), and SR first decreased and then increased (D1 > D3 > D2 or D3 > D1 > D2). GW decreased with increasing N (or planting density). However, under medium N (planting density) levels, GW first increased and then decreased with increasing N (or planting density) (N2 > N1 > N3, D2 > D1 > D3).

Under the same planting density level, GY first increased and then decreased with increasing N application (N2 > N3 > N1). The increase in GY due to N application was more pronounced under low planting density level than under high planting density level, with the best GY increase observed under medium planting density level. Under low and high N levels, GY increased with increasing planting density, whereas under medium N level, GY first increased and then decreased with increasing planting density (D2 > D3 > D1).

The two-year test results showed that the highest GY were obtained under N2D2 (7559.43 kg ha^-^¹ in 2021 and 6528.99 kg ha^-^¹ in 2022). Compared to N1D1, the highest GY increased by 43.87% (5254.49 kg ha^-^¹) in 2021 and 47.03% (4440.63 kg ha^-^¹) in 2022, respectively.

Variance analysis showed that N application rate and planting density had highly significant effects on yield and yield components, with their interaction having a highly significant effect on GW and GY. Correlation analysis showed a highly significant positive correlation between PN and GY (**Figure 3**), indicating that PN was the most important factor in increasing GY.

### 3.4 Rice grain quality

#### 3.4.1 Milling quality and appearance quality

Rice milling quality could be improved with increasing N application rate within a certain range, but increased planting density tended to reduce it. Both higher N application rates and increased planting densities contributed to a decline in the appearance quality of rice (**Table 5**).

**Table 5:** Effects of nitrogen application rate and planting density on rice milling quality and appearance quality.

Year	Treatment	BR (%)	MR (%)	HR (%)	CR (%)	CD (%)
2021	N1D1	81.12 ± 1.07a	72.61 ± 0.13ab	69.94 ± 1.34ab	20.80 ± 1.01d	9.50 ± 0.73d
	N2D1	82.24 ± 0.79a	73.59 ± 0.67a	71.40 ± 0.59a	22.63 ± 2.03cd	11.58 ± 0.43c
	N3D1	81.40 ± 1.13a	71.97 ± 0.91ab	67.70 ± 0.77cd	25.40 ± 1.07bc	13.30 ± 1.17c
	N1D2	80.96 ± 0.32a	72.59 ± 0.80ab	69.38 ± 0.61bc	22.87 ± 2.18cd	11.73 ± 0.49c
	N2D2	82.06 ± 1.12a	73.35 ± 1.18ab	70.19 ± 0.57ab	24.17 ± 1.44bcd	12.30 ± 0.67c
	N3D2	80.77 ± 1.79a	71.53 ± 1.62b	66.61 ± 0.18d	27.30 ± 2.02ab	16.40 ± 1.40b
	N1D3	80.81 ± 0.93a	72.54 ± 1.47ab	68.68 ± 1.74bc	25.97 ± 1.86bc	12.30 ± 0.95c
	N2D3	81.94 ± 1.81a	72.61 ± 0.93ab	69.29 ± 1.06bc	27.78 ± 0.94ab	15.55 ± 1.27b
	N3D3	80.78 ± 1.27a	71.45 ± 0.88b	66.28 ± 0.10d	30.17 ± 2.79a	19.93 ± 0.60a
2022	N1D1	82.20 ± 0.59a	71.24 ± 0.77a	68.59 ± 1.02b	19.65 ± 1.22f	9.04 ± 0.63e
	N2D1	83.55 ± 1.24a	71.87 ± 1.25a	70.42 ± 0.96a	21.74 ± 1.69ef	11.12 ± 0.27d
	N3D1	82.52 ± 1.56a	70.25 ± 1.08a	66.20 ± 0.52c	23.89 ± 0.84cde	12.85 ± 0.76c
	N1D2	82.07 ± 1.10a	71.03 ± 1.60a	68.56 ± 0.28b	21.62 ± 0.92ef	11.13 ± 0.21d
	N2D2	83.33 ± 0.72a	71.86 ± 0.60a	69.41 ± 1.18ab	23.41 ± 0.58de	12.05 ± 0.56cd
	N3D2	82.46 ± 0.57a	70.11 ± 0.68a	66.02 ± 0.31c	26.13 ± 0.95bc	16.21 ± 1.25b
	N1D3	82.12 ± 0.45a	71.23 ± 0.45a	67.62 ± 1.14bc	24.84 ± 1.47bcd	11.94 ± 1.05cd
	N2D3	83.61 ± 0.99a	71.42 ± 1.86a	68.53 ± 1.67b	27.05 ± 1.59b	15.12 ± 0.93b
	N3D3	82.28 ± 1.42a	69.86 ± 1.53a	65.87 ± 0.37c	29.57 ± 0.78a	19.57 ± 1.38a
F-value	Year (Y)	20.143**	22.565**	12.164**	5.446*	2.500ns
	Nitrogen (N)	6.848**	8.850**	59.257**	35.019**	160.360**
	Density (D)	0.286ns	0.563ns	8.502**	48.632**	107.824**
	Y × N	0.085ns	0.024ns	0.093ns	0.071ns	0.027ns
	Y × D	0.097ns	0.050ns	0.469ns	0.060ns	0.016ns
	N × D	0.037ns	0.179ns	0.687ns	0.054ns	8.963**
	Y × N × D	0.044ns	0.038ns	0.108ns	0.035ns	0.031ns

N1, N2 and N3 refer to the different nitrogen fertilizer treatments (90, 150 and 210 kg ha^-1^, respectively). D1, D2 and D3 refer to the different planting density treatments (19.0 × 10^4^, 26.7 × 10^4^ and 40.0 × 10^4^ plants ha^-1^, respectively). BR, MR, HR, CR and CD represent brown rice rate, milled rice rate, head rice rate, chalkiness rate and chalkiness degree, respectively. Different lowercase letters followed the values in the same column mean the significant difference between treatments at *p* < 0.05. ANOVA *p* values and symbols were defined as: * *p* < 0.05; ** *p* < 0.01; ns: *p* > 0.05. The data presented are the mean ± standard deviation, *n* = 3.

As more N was gradually applied, brown rice rate (BR), MR and HR first increased and then decreased (N2 > N1 > N3). No clear pattern in BR was observed with the increased planting density, while MR and HR exhibited a decreasing trend (D1 > D2 > D3). No significant differences were found in BR among the various treatments. Both CR and CD increased with he increased N application rate and planting density (N3 > N2 > N1, D3 > D2 > D1).

The two-year test results showed that the highest MR and HR were obtained under N2D1, while the lowest were obtained under N3D3. Compared to N3D3, MR increased by 2.14 percentage points in 2021 and 2.01 percentage points in 2022, respectively, under N2D1, and HR increased by 5.12 percentage points in 2021 and 4.55 percentage points in 2022, respectively. The lowest CR and CD were obtained under N1D1, while the highest were obtained under N3D3. Compared to N1D1, CR increased by 9.37 percentage points in 2021 and 9.92 percentage points in 2022, respectively, under N3D3, and CD increased by 10.43 percentage points in 2021 and 10.53 percentage points in 2022, respectively.

Variance analysis showed that N application rate significantly impacted all indicators of rice milling and appearance quality. Planting density had a highly significant effect on HR, CR, and CD. Additionally, the interaction between N application rate and planting density had a highly significant effect on CD.

#### 3.4.2 Cooking and eating quality

Rice cooking and eating quality is typically assessed based on 6 key indicators: appearance, mouthfeel, hardness, stickiness, balance, and flexibility value. A comprehensive score (taste value) derived from these indicators provides an overall measure of cooking and eating quality, with higher scores indicating superior quality. Additionally, rice with high peak viscosity (PV) and breakdown viscosity (BV), and low setback viscosity (SV), is considered to have good cooking and eating quality.

Increasing N application rate and planting density both lead to a decrease in RVA characteristic values and cooking and eating quality of rice (**Tables 6**, **7**). As more N was gradually applied, PV, minimum viscosity (MV), BV, and final viscosity (FV) all tended to decrease, while SV increased. The changes in peak time (PeT) and pasting temperature (PaT) were not obvious, and the differences among treatments were not significant. The response of RVA characteristic values to planting density followed a similar trend to that of N application rate, but changes in BV varied over the two-year-test.

**Table 6:** Effects of nitrogen application rate and planting density on the RVA profile characters of rice.

Year	Treatment	PV (RVU)	MV (RVU)	BV (RVU)	FV (RVU)	SV (RVU)	PeT (min)	PaT (°C)
2021	N1D1	267.33 ± 8.35a	202.33 ± 8.56a	65.00 ± 1.53a	278.21 ± 9.49a	10.88 ± 1.62e	6.64 ± 0.04a	76.69 ± 0.75a
	N2D1	249.66 ± 2.42c	187.00 ± 3.50bc	62.66 ± 5.90ab	265.96 ± 0.98bc	16.30 ± 1.63d	6.64 ± 0.11a	77.81 ± 1.59a
	N3D1	233.75 ± 2.73d	174.63 ± 2.45de	59.13 ± 0.74abc	254.12 ± 3.42de	20.37 ± 1.16c	6.63 ± 0.10a	77.82 ± 1.94a
	N1D2	258.17 ± 2.74b	195.54 ± 3.07ab	62.63 ± 5.74ab	273.88 ± 2.32ab	15.71 ± 1.23d	6.58 ± 0.09a	77.40 ± 1.76a
	N2D2	242.25 ± 4.98c	182.67 ± 4.87cd	59.58 ± 2.13abc	261.88 ± 4.40cd	19.63 ± 1.82c	6.67 ± 0.14a	77.44 ± 1.36a
	N3D2	224.80 ± 3.55ef	167.67 ± 4.06e	57.13 ± 0.51bc	255.63 ± 1.29de	30.83 ± 2.34ab	6.64 ± 0.11a	78.12 ± 1.28a
	N1D3	244.92 ± 6.98c	183.96 ± 6.51cd	60.96 ± 2.9abc	266.33 ± 5.89bc	21.42 ± 3.03c	6.57 ± 0.15a	77.13 ± 1.63a
	N2D3	230.96 ± 1.07de	176.79 ± 4.13de	54.16 ± 5.07cd	258.59 ± 1.44cde	27.63 ± 0.46b	6.53 ± 0.09a	77.83 ± 0.56a
	N3D3	218.46 ± 2.70f	170.75 ± 6.11e	47.71 ± 3.41d	251.17 ± 2.58e	32.71 ± 2.63a	6.57 ± 0.12a	77.41 ± 0.14a
2022	N1D1	267.98 ± 6.98a	201.49 ± 5.79a	66.49 ± 1.26a	271.82 ± 6.77a	3.84 ± 3.74b	6.63 ± 0.13a	77.17 ± 1.47a
	N2D1	251.87 ± 6.36bc	187.68 ± 10.86abc	64.19 ± 4.63a	260.19 ± 8.28abc	8.33 ± 2.28ab	6.64 ± 0.12a	78.26 ± 0.40a
	N3D1	234.08 ± 4.42e	170.93 ± 9.45cd	63.15 ± 5.34a	246.94 ± 5.37d	12.87 ± 3.70a	6.62 ± 0.06a	78.23 ± 1.15a
	N1D2	258.52 ± 3.25ab	193.30 ± 8.18ab	65.22 ± 8.68a	267.94 ± 5.42ab	9.42 ± 3.06ab	6.58 ± 0.08a	77.86 ± 0.83a
	N2D2	246.81 ± 6.83cd	183.27 ± 11.26bcd	63.54 ± 8.74a	256.35 ± 9.26bcd	9.54 ± 3.51ab	6.65 ± 0.13a	77.93 ± 1.28a
	N3D2	231.81 ± 3.56e	168.76 ± 9.71d	63.05 ± 7.84a	244.84 ± 6.59d	13.03 ± 3.43a	6.63 ± 0.18a	78.62 ± 1.01a
	N1D3	249.41 ± 7.41bc	181.83 ± 8.00bcd	67.58 ± 4.96a	254.25 ± 7.1cd	4.84 ± 1.65b	6.58 ± 0.04a	77.59 ± 1.17a
	N2D3	238.16 ± 5.85de	174.14 ± 11.44cd	64.02 ± 5.87a	251.13 ± 4.32cd	12.96 ± 4.20a	6.54 ± 0.05a	78.31 ± 0.23a
	N3D3	230.22 ± 4.99e	169.60 ± 8.81cd	60.62 ± 5.11a	243.13 ± 5.17d	12.91 ± 5.60a	6.57 ± 0.09a	77.90 ± 0.88a
F-value	Year (Y)	9.019**	0.300ns	13.989**	23.479**	232.280**	0.009ns	1.944ns
	Nitrogen (N)	136.599**	38.991**	6.046**	50.034**	48.110**	0.081ns	1.785ns
	Density (D)	39.089**	9.446**	2.928ns	10.652**	24.506**	2.843ns	0.187ns
	Y × N	0.862ns	0.032ns	0.659ns	0.214ns	3.928*	0.008ns	0.000ns
	Y × D	1.887ns	0.062ns	2.384ns	0.257ns	12.355**	0.016ns	0.001ns
	N × D	1.789ns	2.000ns	0.619ns	1.572ns	1.524ns	0.536ns	0.493ns
	Y × N × D	0.276ns	0.095ns	0.060ns	0.234ns	1.762ns	0.013ns	0.001ns

N1, N2 and N3 refer to the different nitrogen fertilizer treatments (90, 150 and 210 kg ha^-1^, respectively). D1, D2 and D3 refer to the different planting density treatments (19.0 × 10^4^, 26.7 × 10^4^ and 40.0 × 10^4^ plants ha^-1^, respectively). PV, MV, BV, FV, SV, PeT and PaT represent peak viscosity, minimum viscosity, breakdown viscosity, final viscosity, setback viscosity, peak time and pasting temperature, respectively. Different lowercase letters followed the values in the same column mean the significant difference between treatments at *p* < 0.05. ANOVA *p* values and symbols were defined as: * *p* < 0.05; ** *p* < 0.01; ns: *p* > 0.05. The data presented are the mean ± standard deviation, *n* = 3.

**Table 7:** Effects of nitrogen application rate and planting density on rice cooking and eating quality.

Year	Treatment	TV	Appearance	Mouthfeel	Hardness	Stickiness	Balance	Flexibility value
2021	N1D1	82.00 ± 1.00a	8.10 ± 0.17a	7.33 ± 0.06a	1.60 ± 0.08g	0.52 ± 0.02a	0.19 ± 0.01a	0.86 ± 0.02a
	N2D1	79.67 ± 1.15abc	7.43 ± 0.15cd	6.97 ± 0.06c	1.74 ± 0.07ef	0.43 ± 0.01b	0.17 ± 0.01b	0.86 ± 0.04a
	N3D1	77.33 ± 1.53cde	7.17 ± 0.15def	6.37 ± 0.12f	1.95 ± 0.03bc	0.34 ± 0.02cd	0.16 ± 0.01c	0.87 ± 0.04a
	N1D2	80.67 ± 1.15ab	7.77 ± 0.06b	7.17 ± 0.15b	1.69 ± 0.07fg	0.43 ± 0.02b	0.18 ± 0.01ab	0.86 ± 0.03a
	N2D2	78.67 ± 1.15bcd	7.37 ± 0.06cde	6.67 ± 0.06d	1.81 ± 0.07def	0.36 ± 0.02c	0.17 ± 0.01b	0.86 ± 0.03a
	N3D2	75.33 ± 1.53ef	7.03 ± 0.12fg	6.30 ± 0.10f	2.05 ± 0.10b	0.31 ± 0.01d	0.15 ± 0.01c	0.87 ± 0.02a
	N1D3	79.67 ± 1.53abc	7.53 ± 0.21bc	6.93 ± 0.15c	1.83 ± 0.04cde	0.35 ± 0.01c	0.17 ± 0.01b	0.84 ± 0.02a
	N2D3	76.67 ± 1.53def	7.13 ± 0.15ef	6.53 ± 0.06e	1.94 ± 0.07bcd	0.32 ± 0.02d	0.15 ± 0.01c	0.85 ± 0.02a
	N3D3	74.33 ± 1.53f	6.83 ± 0.21g	6.27 ± 0.06f	2.24 ± 0.08a	0.29 ± 0.01e	0.15 ± 0.01c	0.85 ± 0.04a
2022	N1D1	80.33 ± 0.58a	7.93 ± 0.25a	7.17 ± 0.21a	1.74 ± 0.10e	0.54 ± 0.02a	0.18 ± 0.01a	0.83 ± 0.04a
	N2D1	78.00 ± 1.00b	7.30 ± 0.20bc	6.83 ± 0.25bc	1.87 ± 0.07de	0.43 ± 0.03b	0.17 ± 0.01ab	0.85 ± 0.05a
	N3D1	76.00 ± 1.00c	6.97 ± 0.15d	6.23 ± 0.12e	2.13 ± 0.15bc	0.33 ± 0.02cd	0.15 ± 0.01cd	0.84 ± 0.05a
	N1D2	78.33 ± 0.58b	7.60 ± 0.17b	7.03 ± 0.15ab	1.90 ± 0.12cde	0.42 ± 0.02b	0.16 ± 0.01abc	0.84 ± 0.03a
	N2D2	76.33 ± 0.58c	7.13 ± 0.06cd	6.57 ± 0.06cd	2.02 ± 0.08bcd	0.36 ± 0.01c	0.16 ± 0.01abc	0.85 ± 0.03a
	N3D2	72.67 ± 0.58de	6.83 ± 0.15de	6.10 ± 0.17e	2.17 ± 0.06b	0.32 ± 0.01de	0.14 ± 0.01d	0.85 ± 0.05a
	N1D3	77.33 ± 1.15bc	7.33 ± 0.12bc	6.73 ± 0.23bc	2.04 ± 0.13bcd	0.36 ± 0.02cd	0.16 ± 0.01abc	0.83 ± 0.03a
	N2D3	74.00 ± 1.00d	6.93 ± 0.21de	6.37 ± 0.15de	2.11 ± 0.14bc	0.32 ± 0.02de	0.15 ± 0.01bcd	0.83 ± 0.01a
	N3D3	71.67 ± 0.58e	6.63 ± 0.15e	6.03 ± 0.12e	2.43 ± 0.17a	0.29 ± 0.02e	0.14 ± 0.01d	0.84 ± 0.02a
F-value	Year (Y)	48.014**	17.907**	17.600**	39.842**	0.207ns	25.532**	4.082ns
	Nitrogen (N)	89.503**	109.060**	157.609**	60.983**	213.105**	43.660**	0.365ns
	Density (D)	36.290**	29.088**	25.391**	30.886**	176.363**	11.319**	1.117ns
	Y × N	0.014ns	0.021ns	0.173ns	0.061ns	0.017ns	0.596ns	0.083ns
	Y × D	1.007ns	0.062ns	0.227ns	0.203ns	0.052ns	0.596ns	0.071ns
	N × D	0.910ns	1.339ns	2.345ns	0.570ns	20.561**	2.128ns	0.058ns
	Y × N × D	0.055ns	0.052ns	0.064ns	0.250ns	0.199ns	0.170ns	0.066ns

N1, N2 and N3 refer to the different nitrogen fertilizer treatments (90, 150 and 210 kg ha^-1^, respectively). D1, D2 and D3 refer to the different planting density treatments (19.0 × 10^4^, 26.7 × 10^4^ and 40.0 × 10^4^ plants ha^-1^, respectively). TV represents taste value. Different lowercase letters followed the values in the same column mean the significant difference between treatments at *p* < 0.05. ANOVA *p* values and symbols were defined as: * *p* < 0.05; ** *p* < 0.01; ns: *p* > 0.05. The data presented are the mean ± standard deviation, *n* = 3.

With increased N application and planting density, taste value (TV), appearance, mouthfeel, stickiness, and balance all exhibited a decreasing trend, whereas hardness increased. Changes in flexibility value were minimal and showed no significant differences among treatments.

The two-year test results showed that under N1D1, rice had the highest PV, BV, and TV, and the lowest SV, while opposite trend was observed under N3D3. Compared to N1D1, under N3D3, PV, BV and TV decreased by 22.37%, 36.24% and 10.32%, respectively, in 2021, and by 16.40%, 9.68% and 12.08%, respectively, in 2022. SV increased by 200.64% in 2021 and 236.20% in 2022, respectively.

Variance analysis showed that the effects of N application rate and planting density on PV, SV, and TV were all highly significant. The impact of N application rate on BV was also highly significant. However, the interaction between N application rate and planting density did not significantly affect these indicators. Correlation analysis showed that TV was significantly positively correlated with PV and BV (**Figure 3**), and significantly negatively correlated with SV.

### 3.5 Principal component analysis

Principal component analysis (PCA) of 15 indexes including SPAD value, lipid content, grain yield, effective panicle number, spikelete number per panicle, 1000-grain weight, seed-setting rate, milled rice rate, head rice rate, chalkiness rate, chalkiness degree, taste value, peak viscosity, breakdown viscosity and setback viscosity under different nitrogen application rates and planting densities was used to establish a comprehensive evaluation model.

The comprehensive evaluation results showed that N1D1 got the highest comprehensive score while N3D3 had the lowest (**Table 8**). Rice under N1D1 exhibited the best quality but the lowest yield and lipid content. Conversely, rice under N3D3 had the worst quality but the highest yield and lipid content. N2D2 ranked the third, under which rice achieved the medium quality and lipid content, with the highest yield.

**Table 8:** The scores and rankings under different nitrogen application rates and planting densities.

Treatment	Score	Ranking
N1D1	0.818	1
N2D1	0.617	2
N3D1	-0.404	7
N1D2	0.474	4
N2D2	0.587	3
N3D2	-0.769	8
N1D3	0.213	5
N2D3	-0.280	6
N3D3	-1.254	9

N1, N2 and N3 refer to the different nitrogen fertilizer treatments (90, 150 and 210 kg ha^-1^, respectively). D1, D2 and D3 refer to the different planting density treatments (19.0 × 10^4^, 26.7 × 10^4^ and 40.0 × 10^4^ plants ha^-1^, respectively).

## 4 Discussion

### 4.1 Effects of nitrogen application rate and planting density on rice yield

Rice can effectively utilize light energy resources under optimal planting density and N, ensuring both individual plant growth and coordinated population development (Ge, 2022). Most studies indicate that yield increases with N application up to a certain point, beyond which further increases in N result in decreased yield and some yield components (Dong et al., 2012). Planting density is crucial in establishing a well-structured rice population, closely linked to yield, and significantly influences the PN (Yang, 2003). Excessive N application leads to resource waste and environmental pollution (Jin et al., 2012), while high-density planting intensifies competition for light, temperature, and nutrients among plants, resulting in reduced dry matter accumulation per plant, increased lodging, and higher incidences of pests and diseases (Jensen et al., 2011). Appropriate planting density, combined with reduced N application, can achieve high and stable rice yield (Yang et al., 2019). Research demonstrates that moderate planting density and appropriate N application can enhance effective tillering, promote rice elongation, and improve the population’s photosynthetic capacity, growth rate, and yield (Zhu et al., 2016). Consistent with previous studies, this study found that moderate N application combined with moderate planting density (N2D2) resulted in the highest rice yield (7559.43 kg ha^-1^ in 2021 and 6528.99 kg ha^-1^ in 2022), representing increases of 43.87% (5254.49 kg ha^-1^) in 2021 and 47.03% (4440.63 kg ha^-1^) in 2022 compared to N1D1, respectively.

Research has shown that the net photosynthetic ability of leaves during the grain-filling stage is a critical factor affecting yield, with over 80% of the yield derived from post-heading leaf photosynthesis (Wang et al., 2019). Proper fertilization and planting density significantly influence the net photosynthetic rate and stomatal conductance (Gong et al., 2010). This study found significant interaction effects between N application and planting density on Pn and GY. SPAD and Pn showed strong positive correlations with GY and PN (correlation coefficients of 0.927 and 0.916, respectively). Under N2D3 or N2D2, SPAD and Pn remained high throughout the grain-filling stage, indicating a high yield potential associated with elevated SPAD and Pn values. Effective nutrient utilization and solar radiation interception after heading are crucial for ensuring rice yield (Chen et al., 2015). The interaction between N fertilizer and planting density can enhance and sustain photosynthesis during the grain-filling stage, thereby increasing economic yield (Hou et al., 2019).

### 4.2 Effects of nitrogen application rate and planting density on rice lipid content

The composition of rice includes starch (85-90%), protein (7-12%), lipid (0.2-3%), and trace elements such as minerals and vitamins (Xu et al., 2016). Despite the low lipid content in rice grain, its composition is balanced, and it is rich in physiologically active substances, making it with high nutritional value and health benefits (Yoshida et al., 2010). Notably, the majority of fatty acids in rice grain are high-quality UFAs, including arachidonic acid, linolenic acid, and linoleic acid, which are effective in preventing arteriosclerosis and hypercholesterolemia (Kim et al., 2015). Besides, lipids are a vital class of compounds in living organisms, participating in and regulating numerous biological processes (Zhou et al., 2024). They play a key physiological role in plants’ responses to abiotic stresses, including salt, drought, and temperature stress (Liu et al., 2022; Xue et al., 2024). The synthesis of rice lipid is influenced not only by genetic factors but also by cultivation methods and environmental factors during rice growth. Specifically, N affects carbon-nitrogen metabolism in plants and the synthesis of acetyl-CoA, thereby impacting lipid synthesis (Sharif et al., 2014).

This study found that with increased N application rate, the contents of lipid, FFA, and UFA showed a gradual upward trend, whereas the content of SFA exhibited the opposite trend, consistent with previous research findings (Tu et al., 2020). However, some studies have indicated that increasing N fertilizer during the late growth stage of rice can significantly reduce the lipid content (Zhu et al., 2006). Furthermore, as N application increases, the contents of rice lipid and UFA (oleic acid and linoleic acid) initially increases and then decreases, while the content of SFA (palmitic acid and stearic acid) initially decreases and then increases (Tu, 2019). Therefore, it is hypothesized that significant variations may exist in the changes of lipid and fatty acid content among different rice cultivars under varying N application treatments. N exhibits a regulatory effect on the lipid and fatty acid contents in rice grain, and the amount of fertilizer should be determined based on the sensitivity of different cultivars to N in terms of lipid and fatty acid contents. This approach aims to achieve a synergistic enhancement in lipid content, fatty acid content, yield, and quality.

Research on the impact of planting density on the lipid content in rice grain is relatively limited compared to studies on N application. Planting density significantly influences the distribution of microclimate factors such as photosynthetically active radiation, carbon dioxide, air temperature, wind speed, and relative humidity within the crop population. These effects subsequently influence the composition of the population structure, ultimately reflected in the population’s effective storage energy and yield (Wu, 2021). Studies have shown that planting density significantly impacts the biomass, lipid, and protein contents of cotton seeds. As planting density increases (1.5-8.7 × 10^4^ plants ha^-1^), the lipid content of cotton seeds gradually decreases, demonstrating a highly significant linear negative correlation between lipid content and planting density (Zhu et al., 2010). The synthesis of plant lipid utilizes acetyl-CoA, an intermediate product of carbohydrate metabolism, as a substrate (Zhou et al., 2019). Therefore, lipid synthesis and accumulation are based on the carbohydrates stored in the grains and are regulated by the carbon metabolism pathway. Increased planting density can lead to insufficient light for the population, reducing the photosynthetic rate of leaves and thereby affecting the supply of nutrients to the grains, resulting in a decrease in lipid content (Cai et al., 2018). However, in this study, at low and medium N application levels, lipid content showed an increasing trend with the increase in planting density. At high N application level, lipid content first decreased and then increased with increasing planting density (D3 > D1 > D2). The possible reasons for these observations were as follows: 1) At high N levels, initially, due to the sufficient N supply, plants were not nitrogen-deficient, leading to a preference for protein synthesis over lipid synthesis, thus reducing lipid content (Du et al., 2013); 2) With increasing planting density, the protein content in rice grain decreased, causing more carbon sources to be allocated towards lipid synthesis (Hu et al., 2023); 3) The planting density levels set in this study were not sufficient to reach the inflection point where lipid content begins to decrease, resulting in a continuous increase in lipid content with increasing planting density.

Variance analysis results indicated that N application had a highly significant effect only on the content of FFA, while planting density had a highly significant effect only on the content of UFA. The interaction between these two factors had no significant effect on overall lipid content indicators. Palmitic acid (C16:0), linoleic acid (C18:2), arachidic acid (C18:1), and stearic acid (C18:0) were the predominant fatty acids, accounting for over 90% of the total fatty acid content. However, their responses to N application and planting density did not exhibit a clear pattern. Despite these observations, the differences in lipid and UFA contents between treatments were not significant, whereas the differences in FFA and SFA contents were significant.

These findings suggested that the effects of N application and planting density on the lipid content and fatty acid composition of rice were relatively limited. Future research could explore the combined application of different fertilizers or different fertilizer application methods to study their impact on the lipid content in rice grain.

### 4.3 Effects of nitrogen application rate and planting density on rice quality

The milling quality of rice is a critical determinant of its economic value, with the HR being particularly significant as it decisively influences the market value of paddy rice. Two essential indicators for assessing the appearance quality of rice, which impact its marketability, are the CR and CD (Sharif et al., 2014). Chalkiness refers to the opaque portion of the rice endosperm, and previous research has demonstrated that factors such as cultivar, panicle type, temperature during the grain-filling stage, cultivation method, and fertilizer use all affect the formation of chalkiness in rice (Huo et al., 2012; Long et al., 2016). Studies suggest that with increasing N application, the BR, MR, and HR tend to rise, while the CR and CD tend to decrease (Gong et al., 2013). Additionally, increasing planting density can enhance both the milling quality and appearance quality of rice (Tang et al., 2020). However, some studies indicate that the impact of N application on rice milling quality is relatively small; as N application increases, there is no significant pattern or notable difference in the BR, only a slight increase in the MR and HR, and an increase in the CR and CD (Hu et al., 2017; Yan et al., 2017). With increased planting density, the BR, MR, and HR of machine-sown rice first increase and then decrease, while the CD first decreases and then increases (Sun et al., 2012). This study found that with increasing N application, rice milling quality initially improved and then declined, while increasing planting density deteriorated the milling quality of rice. Both increased N application and planting density resulted in a decline in the appearance quality of rice.

The cooking and eating quality of rice is one of the four major international indicators for evaluating rice quality, reflecting the sensory and physicochemical characteristics of rice during cooking and consumption (Zhai, 2014). Studies have shown that rice with low amylose and protein contents, high PV, high BV, and low SV has better cooking and eating quality (Gao et al., 2010). Research has found that increased N fertilizer application or reduced planting density can increase protein content. Additionally, moderate fertilizer application and appropriate planting density can improve the cooking and eating quality of hybrid japonica rice and conventional japonica rice (Cheng et al., 2011). This study found that both increased N application and planting density led to a decrease in the RVA profile characteristics and cooking and eating quality of rice, differing from previous research conclusions. Previous studies have indicated that higher lipid content in rice grain correlated with better eating quality (Zheng et al., 2018), with FFA and UFA showing a significant positive correlation with taste value (Tu et al., 2020). Conversely, amylose and protein contents are significantly negatively correlated with cooking and eating quality (Jianggu et al., 2016; Zhang et al., 2023). This suggests that the cooking and eating quality of rice is regulated by amylose, protein, and lipid contents. Increasing the lipid content while appropriately reducing the amylose and protein contents can enhance the cooking and eating quality of rice. However, in this study, the contents of lipid and FFA were significantly negatively correlated with the TV, PV, and BV, which differed from previous research findings (Lu et al., 2018; Peng et al., 2022; Tu et al., 2020). This might be due to the different responses of various rice cultivars to N fertilizer and planting density. The effects of N and planting density interactions on rice quality reveal a lack of consistency and even contradictory results, highlighting the diversity of factors influencing rice quality and the complexity of its formation process. Further in-depth research on the mechanisms of these processes is required to better address the issues of optimal fertilization and planting density management for high-quality rice cultivation.

## 5 Conclusion

The lipid content in rice not only has high nutritional value, but its composition and amount also significantly affect rice quality. Thus, the rational regulation of rice lipid content and composition is of great importance for the breeding of high-quality rice and the development of functional rice cultivars. In addition to increasing yield and lipid content in traditional oil crops, utilizing the high-yield characteristics of food crops and enhancing the lipid content of their grains through cultivation methods and other technical means is also a possible approach to converting rice into an alternative oil crop for industry applications. In this study, increasing N application rates and planting density could enhance the lipid and FFA contents in rice grain, although the enhancement effect was relatively limited. Under N3D3, rice exhibited the highest lipid content and moderate yield but the poorest cooking and eating quality. Under N2D2, rice achieved the highest yield, with moderate lipid content and cooking and eating quality. Under N1D1, rice had the best cooking and eating quality but the lowest yield and lipid content. Therefore, for the rice cultivar Koshihikari, applying nitrogen at 150 kg ha^-1^ combined with a planting density of 26.7 × 10^4^ plants ha^-1^ could achieve high yield while also ensuring relatively high lipid content and good cooking and eating quality. This balanced approach offers environmental conservation by reducing the need for excessive fertilizer use and promoting efficient resource utilization.

## Data Availability

The original contributions presented in the study are included in the article/supplementary material. Further inquiries can be directed to the corresponding authors.
